# Supplementary material for: Primary Total Prostate Cryoablation for Localized High-Risk Prostate Cancer: 10-Year Outcomes and Nomograms
Source: Cancers (Basel). 2023 Jul 30;15(15):3873. doi: 10.3390/cancers15153873 (PMC10416842; doi:10.3390/cancers15153873)
Supplement: Supplementary file 1 [file cancers-15-03873-s001.zip › cancers-2537135-supplementary.pdf]

Supplementary materials

Supplementary Figure S1. Flow diagram of patient selection into the study cohort

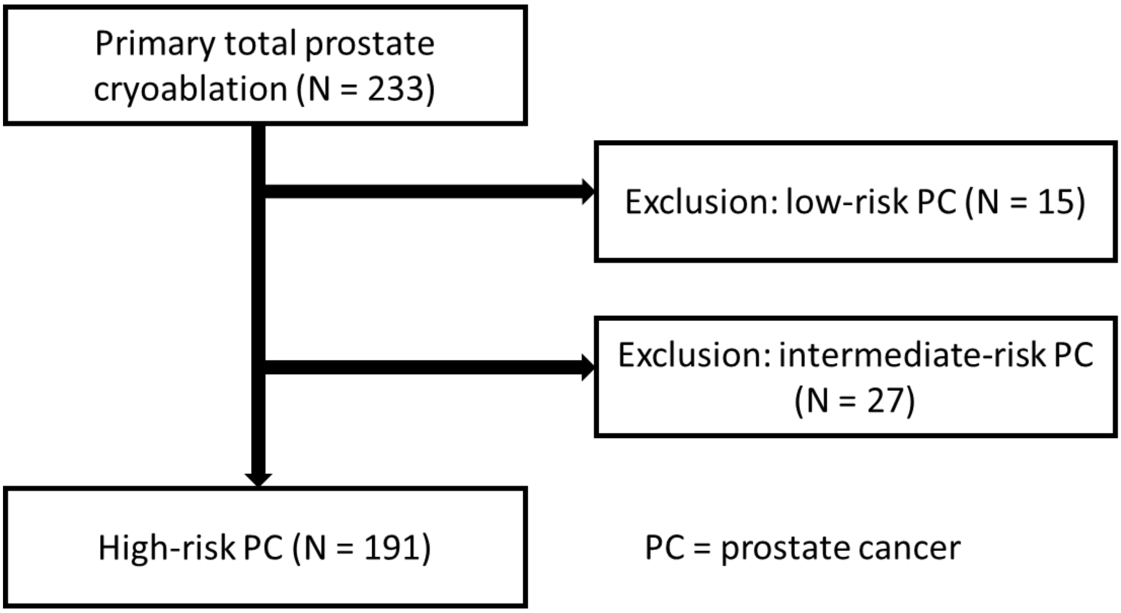

Supplementary Table S1. Demographics and characteristics of the training and validation cohorts

Variables	All		Training cohort		Validation cohort		*p* value
Patient number (n)	191	100%	153	80.1%	38	19.90%	
Mean age (years, SD)	70.3 (7.5)		70.3 (7.6)		70.3 (7.0)		0.955
Mean PSA at diagnosis (ng/ml, SD)	15.4 (10.1)		15.7 (10.4)		14.2 (8.8)		0.419
PSA at diagnosis (ng/ml)							0.352
<10	77	40.31%	59	38.56%	18	47.37%	
10-20	64	33.51%	55	35.95%	9	23.68%	
>20	50	26.18%	39	25.49%	11	28.95%	
Gleason sum							0.838
~6	35	18.32%	27	17.65%	8	21.05%	
3+4=7	61	31.94%	50	32.68%	11	28.95%	
4+3=7	43	22.51%	33	21.57%	10	26.32%	
8-10	52	27.23%	43	28.10%	9	23.68%	
Clinical T stage							0.916
T1-2	49	25.65%	40	26.14%	9	23.68%	
T3a	80	41.88%	63	41.18%	17	44.74%	
T3b-4	62	32.46%	50	32.68%	12	31.58%	

Visible lesions on MRI							0.758
No	32	16.75%	25	16.34%	7	18.42%	
Yes	159	83.25%	128	83.66%	31	81.58%	
Anterior apical tumor							0.907
No	162	84.82%	130	84.97%	32	84.21%	
Yes	29	15.18%	23	15.03%	6	15.79%	
Neoadjuvant hormonal therapy							0.362
No	88	46.07%	73	47.71%	15	39.47%	
Yes	103	53.93%	80	52.29%	23	60.53%	
Adjuvant hormonal therapy							0.546
No	171	89.53%	138	124.32%	33	41.25%	
Yes	20	10.47%	15	13.51%	5	6.25%	
Prostate volume (median in mL, range)	29.9 (12.4)		30.6 (12.9)		27.1 (9.4)		0.126
Cryoprobe number (median, range)	6.7 (1.0)		6.8 (1.0)		6.6 (1.0)		0.414
PSA nadir value (ng/mL)							0.388
<0.01	87	45.55%	72	47.06%	15	39.47%	
0.01-<0.1	66	34.55%	51	33.33%	15	39.47%	
0.1-<0.5	27	14.14%	23	15.03%	4	10.53%	
0.5~	11	5.76%	7	4.58%	4	10.53%	
Time to PSA nadir (weeks)							0.579
<8	71	37.17%	57	37.25%	14	36.84%	
8-<12	76	39.79%	63	41.18%	13	34.21%	
12~	44	23.04%	33	21.57%	11	28.95%	

Supplementary Table S2. The association of PSA nadir value and time to PSA nadir

	PSA nadir value (ng/mL)				
Time to PSA nadir (months)	<0.01	0.01~<0.1	0.1~<0.5	0.5 or more	
< 2	17 (23.9%)	26 (36.6%)	21 (29.6%)	7 (9.9%)	*p* <0.001
2-< 3	40 (52.6%)	31 (40.8%)	2 (2.6%)	3 (3.9%)	
3 or more	30 (68.2%)	9 (20.5%)	4 (9.1%)	1 (2.3%)	

Supplementary Table S3. The association of PSA nadir value and neoadjuvant hormonal therapy

	PSA nadir value (ng/mL)				
Neoadjuvant hormonal therapy	<0.01	0.01~<0.1	0.1~<0.5	0.5 or more	
No	37 (42.1%)	35 (39.8%)	12 (13.6%)	4 (4.6%)	p = 0.538
Yes	50 (48.5%(	31 (30.1%)	15 (15.6%)	7 (6.8%)	
All	86 (45.0%)	67 (35.1%)	27 (14.1%)	11 (5.8%)	

PSA = prostate-specific antigen
